# Exposure to tobacco, alcohol and ‘Junk food’ content in reality TV programmes broadcast in the UK between August 2019–2020

**DOI:** 10.1093/pubmed/fdac046

**Published:** 2022-05-05

**Authors:** Alexander B Barker, Jaspreet Bal, Laura Ruff, Rachael L Murray

**Affiliations:** Department of Psychology, Nottingham Trent University, Nottingham, NG1 4FQ, UK; Academic Unit of Lifespan and Population Health, Faculty of Medicine and Health Sciences, University of Nottingham, Nottingham NG5 1PB, UK; SPECTRUM Consortium, UK; Academic Unit of Lifespan and Population Health, Faculty of Medicine and Health Sciences, University of Nottingham, Nottingham NG5 1PB, UK; Academic Unit of Lifespan and Population Health, Faculty of Medicine and Health Sciences, University of Nottingham, Nottingham NG5 1PB, UK; SPECTRUM Consortium, UK

**Keywords:** alcohol, food and nutrition, smoking

## Abstract

**Background:**

Exposure to alcohol, tobacco and foods high in fat, sugar or salt (HFSS) content in media is a risk factor for smoking, alcohol use and HFSS consumption in young people. We report an analysis of tobacco, alcohol and HFSS content in a sample of reality TV programmes broadcast on TV and video-on-demand services throughout a 1-year period.

**Methods:**

We used 1-min interval coding to quantify content in all episodes of 20 different reality TV programmes between August 2019 and August 2020 and estimated population exposure to a sample of these programmes using viewing data and UK population estimates.

**Results:**

We coded 13 244 intervals from 264 episodes. Tobacco content appeared in 227 intervals (2%) across 43 episodes (2%), alcohol in 5167 intervals (39%) across 258 episodes (98%) and HFSS in 1752 intervals (13%) across 234 episodes (88%). A sample of 15 series delivered ~157.4 million tobacco, 3.5 billion alcohol and 1.9 billion HFSS gross impressions to the UK population, including 24 000, 12.6 million and 21.4 million, to children, respectively.

**Conclusion:**

Tobacco, alcohol and HFSS content are common in reality TV programmes. These programmes deliver exposure to tobacco, alcohol and HFSS imagery, which are a potential driver of tobacco use, alcohol use and HFSS consumption in young people.

## Background

In 2018/2019 in England, smoking and alcohol consumption, respectively, caused an estimated 504 000 and 358 000 hospital admissions.^1^^,^^2^ Obesity is now the third most important risk factor for chronic disease,^3^ and was responsible for 876 000 hospital admissions in 2018–2019.^4^

Since almost all adults who smoke begin during their teenage years,^5^ alcohol consumption in adolescence is associated with a higher risk of consumption in adulthood,^6^ and children and adolescents with obesity typically become adults with obesity;^7–13^ it is important to prevent children and adolescents from experimenting with these behaviours.

There is now strong, causal evidence that exposure to advertising or other tobacco, alcohol and foods high in fat, sugar or salt (HFSS) audio visual content (AVC) in the media, including television programmes, increases subsequent consumption in children and adolescents.^14–30^ Television programme content is widely seen; in 2019 an average person watched over 3 h of television each day.^31^

The Office of Communications (Ofcom) Broadcasting Code^32^ protects under-18 s by restricting depictions of tobacco or alcohol use in programmes made for children, and discouraging the glamorization of tobacco or alcohol use in programmes broadcast before the 9 p.m. watershed^33^ or otherwise likely to be widely seen, heard or accessed by children. Similarly, in 2007, Ofcom and the Advertising Standards Agency introduced regulations prohibiting HFSS foods (junk food), which are a strong risk factor for the development of obesity,^34^ advertising during or adjacent to programmes commissioned for, principally directed at, or likely to appeal particularly to audiences below the age of 16 in the UK.^35^^,^^36^ Further, the 2018 update to the UK Government Childhood Obesity plan proposes to extend the Ofcom prohibition to include programmes broadcast before the 9 pm watershed.^33^^,^^37^

Despite these regulatory controls,^32^ tobacco and alcohol imagery remains prevalent in prime-time television programmes^38–41^ and there is evidence that HFSS content may also appear in popular contemporary media.^42^ We have previously demonstrated that reality television shows contain high levels of tobacco and alcohol imagery,^38^^,^^43–45^ and are often more prevalent than other programme types broadcast within UK prime-time television viewing times.^40^^,^^41^ At the same time, reality TV is likely to appeal to people by providing a form of escapism by presenting an aspirational reality for viewers,^46^^,^^47^ likely to attract younger viewers.^43^^,^^45^ According to Social Learning Theory,^48^ people will imitate the behaviours of influential others, positing the idea that reality TV influences peoples’ behaviours based on what they see on screen; in this way reality TV may be a major driver of unhealthy behaviours.

The current study therefore quantifies tobacco, alcohol and HFSS AVC in a wider sample of reality TV programmes broadcast on UK television throughout a 1-year period, on both broadcast television and video-on-demand (VOD) services, and estimates the population exposure to this content in a sample of programmes.

## Methods

We chose to include all new reality TV programmes (those chronicling people in their daily lives or in fabricated scenarios representing everyday life) which aired on either broadcast TV or VOD services between 1 August 2019 and 1 August 2020 (If a series had started prior to this date, we included the series as a whole).

For every reality TV programmes, we viewed all episodes and measured tobacco, alcohol and HFSS content using 1-min interval coding, a semi-quantitative method, which involved coding each 1-min interval of every episode for the presence of alcohol, tobacco and HFSS content in the following categories^38–41^^,^^43^^,^^49^:

- *Actual Use*: Use of tobacco, alcohol or HFSS on screen by any character, such as seeing a person smoke a cigarette, drink from a pint glass of beer or consuming HFSS food.
- *Implied Use*: Any inferred tobacco, alcohol or HFSS consumption or use without any actual use on screen, such as a verbal reference that a person is going to smoke or drink or eat HFSS food, or a behavioural reference such as removing a cigarette from a packet, holding an alcoholic drink or holding HFSS food.
- *Tobacco paraphernalia/Other Alcohol Reference*: The presence on screen of tobacco, alcohol or HFSS-related materials, such as a lighter, a beer pump/bottle or an HFSS food container or packaging.
- *Brand Appearance*: The presence of clear and unambiguous tobacco, alcohol or HFSS branding, such as seeing a brand on a cigarette packet, beer bottle or food packaging.

Tobacco, alcohol and/or HFSS content were recorded as present in the 1-min interval if there was one appearance of any category. More than one category could be coded in a single interval, for example both alcohol and tobacco use. Multiple instances of the same category in the same interval were recorded as one event, but if the same event overlapped two intervals, this was coded as two separate events. One-third of the recorded footage was coded separately by two authors to ensure accuracy and reliability in the coding method.

HFSS content was checked against the Ofcom ‘Big 6’ categories of food,^36^ HFSS brands were recorded and checked against the Nutrient Profiling Tool^50^ to ensure that the brand was in fact an HFSS product. HFSS brands were also included if they are associated with HFSS food, such as food delivery services (e.g. Deliveroo) or food establishments (e.g. McDonalds), as well as brands which were not necessarily for HFSS products but the overarching brand is (e.g. Diet Coca Cola), as key features of the brand are also used and this product could act as an ‘alibi brand’.^51–53^

We estimated the UK audience population exposure using viewing data from *Digital.I*^54^ and UK mid-year population estimates for 2020 combined with numbers of tobacco, alcohol and HFSS appearances to estimate gross and per capita impressions by age group, using previously reported methods.^43^^,^^45^^,^^55^^,^^56^ The method involves combining viewership (obtained from viewing figures) with the number of tobacco/alcohol/HFSS appearances per episode to provide gross impressions (the estimated number of tobacco/alcohol/HFSS exposures delivered). Dividing gross impressions by population mid-year estimates provided per capita impressions— the estimated number of tobacco/alcohol/HFSS impressions delivered to each person. Both gross and per capita impressions were computed by age group. The confidence level was set to 95%.

## Results

In total, we recorded 13 244 1-min intervals of footage (220.7 h), across 264 episodes from 20 different reality TV programmes (Table 1). Netflix was the only VOD service which included programming which was coded.

**Table 1:** Summary of included programmes

Title	Series No.	TV/Vod	Channel	Country of Origin	No. of episodes	Date first episode aired in the UK
Absolutely Ascot^*^	2	TV	ITVBe	UK	8	22^nd^ September 2019
Ferne McCann First Time Mum^*^	4	TV	ITVBe	UK	3	30^th^ October 2019
Geordie Shore^*^	20	TV	MTV	UK	10	29^th^ October 2019
Gemma Collins: Diva Forever^*^	2	TV	ITVBe	UK	5	7^th^ August 2019
Gemma Collins: Diva on Lockdown^*^	1	TV	ITVBe	UK	3	26^th^ April 2020
Ibiza Weekender^*^	6	TV	ITV2	UK	10	5^th^ Jan 2020
I’m a celebrity, get me out of here!^*^	19	TV	ITV	UK	22	17^th^ November 2019
Keeping up with the Kardasians^*^	17	TV	E!	US	12	15^th^ September 2019
Love is Blind	1	VOD	Netflix	US	10	13^th^ February 2020
Love Island: Australia^*^	2	TV	ITVBe	Australia	29	14^th^ October 2019
Love Island: Winter		TV	ITV2	UK	36	12^th^ January 2020
Made in Chelsea^*^	18	TV	E4	UK	11	2^nd^ September 2019
Made in Chelsea	19	TV	E4	UK	6	23^rd^ March 2020
Married at First Sight: Australia	4	TV	Channel 4	Australia	26	29^th^ June 2020
Spencer, Vogue and Wedding two^*^	1	TV	E4	UK	4	21^st^ October 2019
The Circle^*^	2	TV	Channel 4	UK	22	24^th^ September 2019
The Real Housewives of Cheshire^*^	10	TV	ITVBe	UK	9	9^th^ September 2019
The Only Way is Essex^*^	25	TV	ITVBe	UK	11	1^st^ September 2019
Teen Mom UK^*^	6	TV	MTV	UK	8	24^th^ July 2019 (Mid-season at start date of the study)
Too Hot to Handle	1	VOD	Netflix	US	8	17^th^ April 2020

^*^Included in the population exposure estimate

### Tobacco

Tobacco content occurred in 227 intervals (2%) across 43 episodes (2%). Actual tobacco use was seen in seven intervals across four episodes, all featuring cigarette smoking. Implied use was seen in 24 intervals across 16 episodes. Tobacco paraphernalia was seen in 211 intervals across 36 episodes, with the most common being ash trays which were seen in 137 intervals, followed by lighters, cigarette packets, matches and other content which were seen in 44, 19, 2 and 9 intervals, respectively. In addition, ‘no smoking signs’ were seen in 27 intervals. Tobacco brands were seen in two intervals, with the brands ‘Camel’, ‘Lucky Strike’ and ‘Marlboro’ each being seen once on cigarette packets.

### Alcohol

**Fig. 1:**
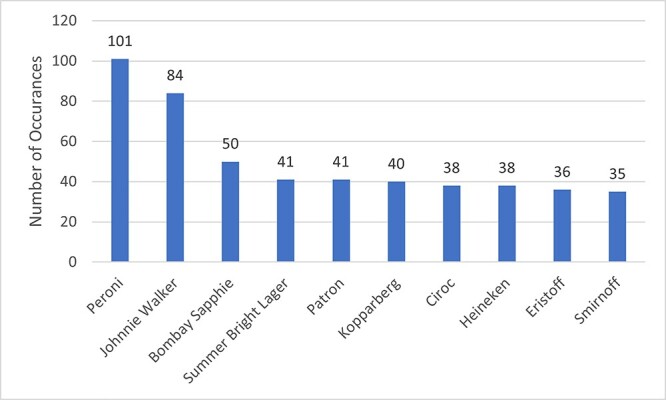
The top 10 most commonly occurring alcohol brands.

Alcohol content was seen in 5167 intervals (39%) across 258 episodes (98%). Actual alcohol use was seen in 966 intervals across 212 episodes, with wine/champagne the most common type (consumed in 582 intervals). Implied alcohol use was seen in 4177 intervals across 250 episodes, with the most common being a person holding a drink (3935 intervals). Alcohol paraphernalia was seen in 2369 intervals across 240 episodes, with the most commonly seen content being beer pumps/bottles seen in 1989 intervals. Alcohol branding was seen in 479 intervals across 122 episodes. In total, 149 different brands were seen with the most seen brands being ‘Peroni’ which was seen 101 times across 58 intervals and ‘Johnnie Walker’, which was seen 84 times across 15 intervals (See Fig. 1).

### HFSS

**Fig. 2:**
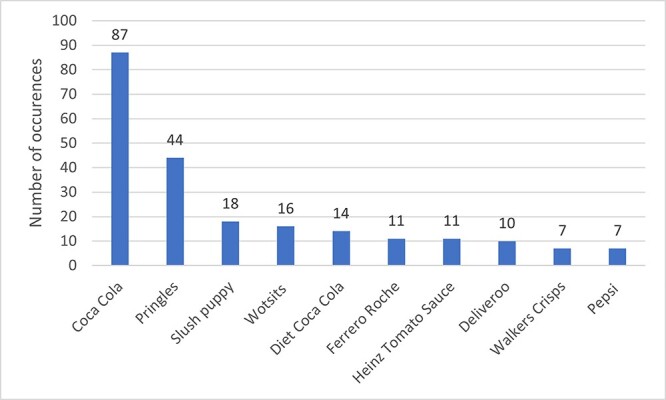
The top 10 most commonly occurring HFSS brands.

HFSS content was seen in 1752 intervals (13%) across 234 episodes (88%). Actual HFSS consumption was seen in 288 intervals across 137 episodes, with the most common food category consumed being confectionary in 73 intervals. Implied consumption was seen in 897 intervals across 180 episodes. Other HFSS content was seen in 924 intervals across 204 episodes with actual food being shown on screen the most frequently—399 intervals. HFSS branding was seen in 333 intervals across 102 episodes; in total 93 brands were seen with the most common being Coca Cola which appeared 87 times across 79 intervals, whereas Diet Coke was only seen 14 times (See Fig. 2).

**Table 2:** Comparison to previous study

	Current study (13 244 intervals)	Previous study (5219 intervals, (Barker et al., 2020))	Significance (*P*)
Any Tobacco Content	227	110	0.75
Any Alcohol Content	5167	2212	0.00

#### Comparison with previous study

Compared with our previous analysis of reality TV programmes,^45^ the proportion of intervals containing alcohol content was significantly higher in the previous study (See Table 2).

#### Difference between services/countries

Significantly more tobacco (any content, use, implied, use, paraphernalia and branding) and HFSS content (any content, use, implied use, other content and branding) was seen on broadcast TV than on programmes viewed on Netflix. While there was no significant difference in the amount of any alcohol content and implied use shown, there were significant differences with use, branding and other content, with a higher proportion of intervals containing content shown on broadcast TV (See Supplementary file 1).

While there was no significant difference in the amount of tobacco use and branding shown between countries, significantly more tobacco content, implied use and paraphernalia were shown in programmes from the UK than that from the USA or Australia. Significantly more intervals containing any alcohol content, alcohol use, implied alcohol use, alcohol branding, HFSS content, HFSS use, HFSS implied use, HFSS other content and HFSS branding in programmes were made in the UK than in the USA or Australia (Supplementary file 1).

#### Population Exposure Estimate

A convenience sample of programmes, based upon available funding and availability of data, was selected to be a part of a population exposure estimate. The following 15 programmes were selected for inclusion and consisted of 165 episodes out of the 264 from the original sample (See Table 1).

We estimate that the 165 episodes delivered 157.4 million tobacco gross impressions to the UK population, including 9.6 million to children aged under 16. Tobacco impressions per capita were highest in the 25–34 age group (average 4.96 (95% CI 4.48–5.47)). Children received on average 0.74 (95% CI 0.57–0.94) per capita impressions. There were 569 000 gross impressions of branded tobacco products, including 24 000 to children under the age of 16.

The sample delivered an estimated 3.5 billion alcohol gross impressions to the UK population, including 197.3 million to children aged under 16. Alcohol impressions per capita were highest in the 25–34 age group (average 77.87 (95% CI 70.93–84.79)). Children received on average 15.55 (95% CI 12.84–18.27) per capita impressions. There were 238.8 million gross impressions of branded alcohol products, including 12.6 million to children under the age of 16.

The sample delivered an estimated 1.9 billion HFSS gross impressions to the UK population, including 136.6 million to children aged under 16. HFSS impressions per capita were highest in the 65+ age group (average 35.22 (95% CI 32.12–38.33)). Children received on average 29.15 (95% CI 24.17–34.16) per capita impressions. There were 282.3 million gross impressions of branded HFSS products, including 21.4 million to children under the age of 16.

## Discussion

### Main findings of the study

The current study demonstrates that in reality TV programmes shown between July 2019 and August 2020, tobacco imagery was rarely seen. In contrast, alcohol and HFSS imagery was particularly prominent occurring in 98% and 88% of episodes, respectively. The tobacco, alcohol and HFSS content of these programmes generates substantial population exposure, of the order of millions of impressions for tobacco and billions for alcohol and HFSS. While this included a relatively small exposure for tobacco content to children under 16, these included millions of alcohol and HFSS impressions to children under 16 years of age.

### What is already known on this topic

The initiation of smoking, alcohol use and HFSS food choices at a young age is a risk factor for dependence and continued use in later life. There is now strong evidence to suggest that exposure to advertising and other content in the media increases subsequent use in adolescents. Previous studies have found that tobacco and alcohol content is frequently shown on UK television, with reality TV programmes showing a large amount of content and exposing millions of young people to this.^45^

### What this study adds

Our study provides further evidence that reality TV programmes are a significant source of exposure of children to tobacco, alcohol and HFSS imagery. The programmes included in this analysis represent a variety of different reality TV programmes, including international formats shown on UK television, and broadcast on a range of both free and paid-for TV channels.

While previously suggested that programmes on VOD services may depict more content than broadcast TV,^57^ the current study found that reality TV programmes broadcast on TV showed significantly more content than reality TV programmes broadcast on Netflix; reality TV programmes are however a new venture for VOD services. Both of the series explored in the current study were the first season of the programme, and due to the difference in regulations covering Netflix^57^^,^^58^ represent a potential source of exposure and should continue to be explored.

The current study suggests that the amount of content shown in reality TV programmes is dependent on the country in which the programme is made, with significantly more content, particularly brands, being shown in programme made in the UK than in the USA and Australia. While the Ofcom Broadcasting Code prevents paid-for alcohol product placement, brands can appear in programmes if they are considered ‘editorially justified’ and were acquired at no significant value, with no providers being paid for the product.^32^^,^^59^ We have previously suggested that alcohol producers may be using reality TV programmes to circumvent the Ofcom Broadcasting code;^45^ the current study continues to show that alcohol brands are receiving widespread exposure to this content through these programmes. In the UK, product placement of HFSS products is not currently prohibited, which may lead to an increase in branded content featuring in programmes rather than advertisements as the proposed watershed ban on HFSS advertisements is introduced, similar to what has been previously seen with alcohol and tobacco content.^43–45^ We also saw evidence of food delivery services using programmes to promote their services, which may represent a way to continue to promote these services despite an advertisement ban. Despite a ban on paid-for alcohol product placement, alcohol brands were more regularly featured in reality TV programmes than HFSS brands, which are not currently prohibited, suggesting that the current restrictions are ineffective at preventing brand exposure in programmes.

While our previous analyses have focussed on a single show, or a small sample of shows shown during a small period of time, the current study shows that tobacco, alcohol and HFSS content are shown on a variety of different reality TV programmes throughout the year, appealing to different demographics, potentially highlighting that young people may be continually exposed to this content through different shows.

We found evidence of cross-border marketing in the current study, with brands being seen almost exclusively in specific programmes. By featuring these products in reality TV programmes, brands are gaining exposure not only to a domestic audience but also internationally, with the potential to bypass country-specific regulation on alcohol marketing.

The current study continues to show that reality TV programmes are viewed by young people and expose them to alcohol and tobacco content, contrary to section 1.10 of the Ofcom Broadcasting Code.^32^ Furthermore, the Ofcom Broadcasting Code does not cover HFSS content, however, section 1, which includes controls on tobacco and alcohol, is led by the following principle: ‘To ensure that people under 18 are protected’. As exposure to HFSS content is causally linked to HFSS consumption and therefore the morbidity and mortality are associated with this throughout the lifespan, the current regulation regarding the inclusion of this content in television programmes should be reviewed.

The obesity policy context in the UK is currently changing with HFSS advertisements moving until after the 9 pm watershed,^33^ however, this does not include HFSS programme content or exempt content. The current study highlights that HFSS content and branding occurs in programmes and young people will likely continue to be exposed to this content. Stricter controls are needed to prevent this as-yet unregulated promotion of HFSS foods through programme content; a ban on advertisements should include brands shown in programmes.

### Limitations of this study

The current study explored content in a large sample of programmes across a large period of time; however, the authors acknowledge that they may not have explored content in every programme shown during this period due to the wide variety of channels available in the UK. A sample of programmes coded were included in population exposure calculations; the true exposure to content in the total amount of programmes coded is likely higher than that reported; furthermore, these programmes are often repeated on other channels and these programmes are also available in countries other than the UK. The study was ongoing during the 2020 Covid-19 pandemic and the subsequent ‘lockdown’ in the UK. As a result, series finished early, such as Made in Chelsea season 19, and programmes were adapted to continuing with the ongoing situation, such as Gemma Collins: Diva on Lockdown. This likely affected the number of programmes included in the 1-year period. There is also evidence that the average TV viewing time increased as a result of the Covid-19 pandemic, with an increase in viewing VOD services;^60^ two of the reality TV programmes featured in the current study were released on Netflix and likely led to widespread exposure. Unfortunately, these programmes were not included in the population exposure, due to the additional cost of obtaining viewing figures for VOD services. Future studies should explore the exposure to content on VOD services.

Reality TV programmes feature alcohol and HFSS content. These programmes are widely viewed and seen by young people and due to the nature of reality TV, with its inspirational role models, they are likely influencing drinking and food consumption choices in young people. The current rules and regulations are not sufficient to prevent this exposure to potentially harmful content and need revision to prevent this exposure.

## Declaration of interests

None to declare.

## Funding

This work was supported by Cancer Research UK [C63710/A27908]. The funders had no role in the study design, data collection and analysis, decision to publish or preparation of the manuscript.

## Supplementary Material

Supp_file_1_fdac046

## Notes

**Alexander B Barker**, Lecturer in Psychology

**Jaspreet Bal**, Research Assistant

**Laura Ruff**, Medical Student

**Rachael L Murray**, Professor of Population Health
