# Supplementary material for: Exposure to tobacco, alcohol and ‘Junk food’ content in reality TV programmes broadcast in the UK between August 2019–2020
Source: J Public Health (Oxf). 2022 May 5;45(2):287–94. doi: 10.1093/pubmed/fdac046 (PMC10273349; doi:10.1093/pubmed/fdac046)
Supplement: Supp_file_1_fdac046 [file supp_file_1_fdac046.docx]

Supplementary File 1: Differences in the number of intervals containing content between Services and Country of Origin

|  |  | Service | | | Country of Origin | | | |
| --- | --- | --- | --- | --- | --- | --- | --- | --- |
| Product | Data category | Netflix  (890 intervals) | TV (12354 intervals) | Significance (p) | UK (8679 intervals) | USA (1410 intervals) | Aus (3155 intervals) | Significance (p) |
| Tobacco | Any Content | 0 | 227 | <.01 | 224 | 1 | 2 | <.01 |
|  | Use | 0 | 7 | <.01 | 7 | 0 | 0 | .22 |
|  | Implied Use | 0 | 24 | <.01 | 23 | 0 | 1 | <.01 |
|  | Paraphernalia | 0 | 211 | . <.01 | 209 | 1 | 1 | <.01 |
|  | Branding | 0 | 2 | <.01 | 2 | 0 | 0 | 1.0 |
| Alcohol | Any Content | 329 | 4838 | .201 | 3640 | 451 | 1076 | <.01 |
|  | Use | 77 | 889 | .031 | 552 | 100 | 314 | <.01 |
|  | Implied Use | 269 | 3908 | .390 | 2973 | 356 | 848 | <.01 |
|  | Other Content | 199 | 2170 | <.01 | 1575 | 243 | 551 | .561 |
|  | Branding | 15 | 464 | <.01 | 243 | 18 | 218 | <.01 |
| HFSS | Any Content | 37 | 1715 | <.01 | 1405 | 184 | 163 | <.01 |
|  | Use | 8 | 280 | <.01 | 220 | 21 | 47 | <.01 |
|  | Implied use | 15 | 882 | <.01 | 736 | 102 | 59 | <.01 |
|  | Other Content | 23 | 901 | <.01 | 744 | 107 | 73 | <.01 |
|  | Branding | 5 | 328 | <.01 | 274 | 28 | 31 | <.01 |
